# Association between daily step counts and physical activity level among Korean elementary schoolchildren

**DOI:** 10.20463/jenb.2016.09.20.3.8

**Published:** 2016-09-30

**Authors:** Jonghoon Park, Kazuko Ishikawa-Takata, Sangjik Lee, Eunkyung Kim, Kiwon Lim, Hyungryul Kim, In-Sook Lee, Shigeho Tanaka

**Affiliations:** 1 Department of Physical Education, Korea University, Seoul Republic of Korea; 2 Department of Nutritional Education, National Institute of Health and Nutrition, Tokyo Japan; 3 Division of Physical Education, Uiduk University, Gyeongju Republic of Korea; 4 Department of Food and Nutrition, Gangneung-Wonju National University, Gangneung Republic of Korea; 5 Department of Physical Education, Konkuk University, Seoul Republic of Korea

**Keywords:** Step counts, PAL, Children

## Abstract

**[Purpose]:**

The purpose of the current study was to investigate steps per day (steps/d) and physical activity level (PAL) in Korean elementary school children having normal weight (normal-weight). We also clarified whether a gender difference exited between steps/d and PAL.

**[Methods]:**

Children aged 9 to 12 y were recruited from two elementary schools located in different urban districts in Korea. The present study included 33 Korean children, of which 18 were normal-weight boys and 15 were normal-weight girls. During the same 1 week study period under free-living conditions the total energy expenditure (TEE) and step counts were estimated using the doubly labeled water (DLW) method and an accelerometer, respectively. We calculated PAL as the TEE/ resting metabolic rate.

**[Results]:**

The range of PAL was 1.25 – 1.93 with a mean value of 1.57. None of the variables of energy expenditure was significantly different by sex. However, steps/d were significantly higher in boys than in girls. When adjusting regression analysis by gender, steps/ d were positively associated with PAL among all subjects (r = 0.56, P < 0.01). Furthermore, steps/d were positively associated with PAL in boys (r = 0.68, P < 0.01), but not in girls (r = 0.27, P = 0.34).

**[Conclusion]:**

Our results suggest that locomotive activity may be the main contributor to the individual PAL differences for elementary school boys, while non-locomotive activity may be the main contributor for elementary school girls.

## INTRODUCTION 

Physical inactivity during youth is related to obesity and health-related risks, such as cardiovascular diseases and diabetes^1^^-^^3^, while increasing physical activity (PA) can reduce the risks associated with these diseases^4^^,^^5^. Recently, there has been much evidence of a considerable decrease in PA in elementary schoolchildren. In Korea, steps per day (steps/d) in urban elementary school children (9-11 years old) were on average 11,165 and 10,708 in boys and girls, respectively^6^. The steps/d in the Yamauchi *et al*. study^6^ were below the criterion of 15,000 and 12,000 steps per day for boys and girls, respectively, where this criterion was suggested by Tudor-Locke *et al*.^7^. Lower steps/d are also observed among elementary school children in other countries^8^^-^^10^. Itoi *et al*.^11^ recently reported that steps/d in urban elementary school children (11-12 years old) in Japan considerably decreased during a decade (1999 to 2009) (20,832 vs. 12,237 steps per day in boys and 16,087 vs. 10,748 steps per day in girls) with a concomitant decrease in physical activity level (PAL, the ratio of total energy expenditure (TEE): resting metabolic rate (RMR)) assessed by accelerometry. This result suggests that childhood PAL, specifically steps for daily physical activities, might have decreased over the past few decades. 

In studies of Western children using the doubly labeled water (DLW) method, the most accurate method for measuring TEE in free-living conditions^12^^,^^13^, the DLW-measured PAL values were within range of the mean, i.e., 1.61-1.74, in elementary school children^14^^-^^16^. In these studies, the PAL values did not differ between boys and girls. In Asia, Krishnaveni *et al*.^17^ reported that DLW-measured PAL was 1.5 and 1.4 in boys and girls (aged 8 to 9), respectively, but they did not provide information on whether they were elementary school children or not. To our knowledge, PAL data using the DLW method for Asian normal-weight boys and girls in elementary school have not been reported yet. 

In our previous study^18^ using the DLW method, steps/d was positively associated with PAL in adult male (r = 0.64, P = 0.02), but not in adult female subjects (r = 0.08, P = 0.70). This indicates that the relationship between steps/d and PAL may be different between sexes. However, it remains unknown whether the result of the sex difference between steps/d and PAL among adults could be applicable to children who are attending elementary school. In addition, children have different patterns of physical activities compared with adults, as the normative tempo of children’s physical activity is one of rapid change from one short activity event to another, from one level of intensity to another^19^. 

The current study aimed to investigate the relationship between steps/d and DLW-measured PAL in Korean normal-weight elementary school children. Furthermore, we clarified whether a gender difference exits between the steps/d and PAL. We hope that information from the present study will help to understand the role of step counts as a criterion of maintaining optimal heath among elementary school children. 

## METHODS 

### Subjects 

Children aged 9 to 12 y were recruited from two elementary schools located in urban districts of Pohang and Gangneung city, Korea. We announced this project to all of the teachers of the school to recruit participants according to the following criteria: (a) in good health, (b) not involved in hard physical activity, such as athletic activity, and (c) living in their home prefecture for 2 weeks before and during the study. The participants’ parents were also informed that the study concerned the measurement of the daily physical activity and food intake of children. A total of 74 subjects (49 boys and 25 girls) were recruited in the present study. 5 subjects (4 boys and 1 girls) failed to produce urine samples or produce sufficient data for DLW measurement. Additionally, 26 overweight boys and 7 overweight girls were excluded in the present study analysis because the number of overweight or obese girls had not been sufficient for statistically significant analysis. Finally, except 1 boy and 2 girls, who could not produce data on their step counts, 18 boys and 15 girls were included in the present study. This study was conducted according to the guidelines laid down in the Declaration of Helsinki and all procedures involving human subjects were approved by the Ethical Committee of the National Institute of Health and Nutrition in Japan (Pohang study) and by the Ethical Committee of Gangneung-Wonju National University (Gangneung study). Written informed consent was obtained from all subjects and their parents. This study was also approved by the School Board Officials prior to beginning the studies. 

### Experimental procedures 

This study was performed in April of 2010 (spring season) in Pohang city (Pohang study), Korea and in November of 2013 (fall season) in Gangneung city (Gangneung study), Korea. The first semester of elementary school was from March to July and the second semester was from September to February. On the day before assessment of physical activity began, urine samples (baseline) were collected and body weight and height measured. A single dose of DLW was then administered orally to each subject. After this dose was administered, the participants were requested to provide urine samples 5 additional times on the following 8 days at the same time of the day. Their resting metabolic rate (RMR) was measured in the early morning, and 12 hours or longer after the last meal during the study period. Subjects were instructed to wear an accelerometer during the study period and also asked to keep a dietary record. 

### Measurement of energy expenditure and body composition 

The single dose of DLW was composed of approximately 0.06 g/kg body weight of ^2^H_2_O (99.8 atom%, Cambridge Isotope Laboratories, MA, USA) and 1.4 g/kg body weight of H_2_^18^O (10.0 atom%, Taiyo Nippon Sanso, Tokyo, Japan). TEE was measured using the DLW method as described previously^20^. Calculation of TEE (kcal/d) was performed using a modified Weir’s formula^21^ based on the CO_2_ production rate and respiratory quotient (RQ). The mean food quotient (FQ) (0.87±0.02) calculated from a 3-day dietary record was used instead of RQ. This assumes that under conditions of perfect nutrient balance the FQ must equal the RQ^16^^,^ ^22^. PAL was estimated by dividing TEE by RMR. 

### Step counts 

The Active style Pro (Omron Health Care Co., Ltd., Kyoto, Japan) was used for the measurement of step counts. The Active Style Pro has proven reliable for estimating step counts^20^^,^ ^23^. Subjects wore the accelerometer on the right side of the waist. The accelerometer was monitored continuously for 7 days including 5 weekdays and 2 weekend days. Non-wearing time for the Active Style Pro was based on the Active Style Pro counts. We excluded days in which there were less than 600 minutes of wearing time during daytime^24^. Data with periods of zero values of more than 60 min were considered non-wearing time^25^. Subject data were considered to be valid if the accelerometer data were counted for at least three weekdays and one weekend day^25^ : all subjects met the criteria. 

### Other measurements 

Body weight was measured to the nearest 0.1 kg and height was measured to the nearest 0.1 cm in individuals wearing the very light clothing, namely with underwear and no shoes. BMI was calculated as body weight (kg) divided by the square of body height (m^2^). The measurement of RMR was performed using a Douglas bag (Pohang study) and open-circuit indirect calorimetry (Gangneung study, a ventilated hood system, TrueOne2400 Parvo Medics, USA), as described previously^26^^,^ ^27^. The dietary intake of each child was assessed by the 3-day dietary records that were maintained by each child with help from his/her parents for 3 consecutive days (2 weekdays and 1 weekend day). Each day, well-trained dietitians checked the record they received to find omissions or errors and corrected them by asking questions of each participant. Food intake was calculated using the CAN-pro 3.0 (Computer Aided Nutritional Analysis version 3.0 for professional) program by the Korean Nutrition Society. Calculated mean FQ from the 3-day dietary record was used when calculating TEE. 

### Statistics 

All values are presented as means ± SD. An unpaired t-was used for the comparison of groups after checking normal distributions using Levene’s test. The relationship between step counts and PAL was examined using Pearson’s correlation coefficients (r). A value of p < 0.05 was considered statistically significant. All statistical treatments were done using the SPSS (IBM SPSS statistics version 23; SPSS Inc., Chicago, IL, USA). 

## RESULTS 

Physical characteristics of the participants are shown in Table 1. There was no difference between boys and girls in the data of physical characteristic such as age, weight, height and BMI. 

**Table 1:** Physical characteristics of subjects

	Boys (n = 18)	Girls (n = 15)	p value
Age (y)	10.6 ± 1.0	11.1 ± 0.7	0.15
Weight (kg)	37.6 ± 7.6	39.8 ± 7.0	0.38
Height (m)	1.44 ± 0.1	1.46 ± 0.1	0.41
BMI	18.0 ± 2.5	18.5 ± 1.8	0.53

All values are means ± SD. BMI, body mass index (kg/m^2^).

**Figure 1.**
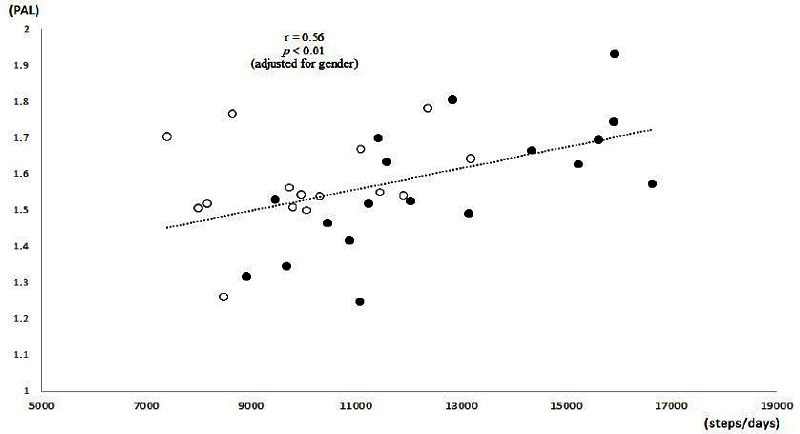
Relation between step counts per day and physical activity level (PAL). PAL = total energy expenditure / resting metabolic rate. TEE was measured by the doubly labeled water method. Filled circles: normal-weight boys, Opened circles: normal-weight girls. The association between steps/d and PAL was shown in Figure 1. Steps/d were positively associated with PAL among all subjects (r = 0.47, P < 0.01; PAL = 0.00003*steps/d + 1.236). When analyzing the data in boys and girls, respectively, steps/d were positively associated with PAL in boys (r = 0.68, P < 0.01), but not in girls (r = 0.27, P = 0.34). When adjusting the regression analysis by gender, r was 0.56 (P < 0.01; PAL = 0.00004015*Steps/d + 0.108*Gender + 0.954 [Gender: ‘boy=1, girl=2’; Steps/d: continuous variables]).

The variables of energy expenditure and daily step counts are shown in Table 2. The range of PAL was 1.25 – 1.93 with a mean value of 1.57 (1.57 vs. 1.57 in boys and girls, respectively). Neither variables of energy expenditure were significantly different between the groups. Steps/d were significantly higher in boys (mean 12,578) than in girls (mean 10,031) (P < 0.01). There were 5 boys who walked above 15,000 steps per day, but no girls. The association between steps/d and PAL was shown in Figure 1. Steps/d were positively associated with PAL among all subjects (r = 0.47, P < 0.01; PAL = 0.00003*Steps/ d + 1.236). When adjusting regression analysis by gender, r was 0.56 (P < 0.01; PAL = 0.00004015*Steps/d + 0.108*Gender + 0.954 [Gender: ‘boy=1, girl=2’ Steps/d: continuous variables]). When analyzing the data in boys and girls, respectively, steps/d were positively associated with PAL in boys (r = 0.68, P < 0.01), but not in girls (r = 0.27, P = 0.34). 

**Table 2:** Energy expenditure and step counts

	Boys (n = 18)	Girls (n = 15)	p value
TEE (kcal/d)	1986 ± 384	1906 ± 331	0.53
RMR (kcal/d)	1268± 205	1214 ± 193	0.45
PAL	1.57 ± 0.18	1.57 ± 0.13	0.92
Step counts (counts/d)	12578 ± 2479	10031 ± 1719	< 0.01^*^

Data are expressed as mean ± SD or median (range). BMI, body mass index (kg/m^2^); TEE, total energy expenditure; RMR, resting metabolic rate; PAL, physical activity level (=TEE/RMR); RMR and TEE were assessed by a Douglas bag and doubly labeled water method, respectively.

^*^indicates a significant difference between two groups.

## DISCUSSION 

To our knowledge, this is the first report to analyze the relationship between steps/d and DLW-measured PAL under free-living conditions in elementary school children. The principal findings of the present study were as follows: DLW-measured PAL did not differ between normal-weight boys and girls. However, boys had more steps/d compared with girls. In addition, a positive relationship between steps/d and PAL was observed in boys, but not in girls. 

In the present study, the PAL value was a mean of 1.57 in Korean normal-weight elementary school children. The mean PAL values in the present study were slightly lower compared with Western elementary school children whose DLW-measured PAL values were within range of the mean of 1.61-1.74^14^^-^^16^. In addition, the lack of a significant difference in PAL between normal-weight boys and girls in the present study was consistent with previous studies reporting DLW-measured PAL among elementary school children aged 7 to 12 in western counties^14^^-^^16^. 

Despite the lack of a significant difference in PAL between boys and girls in the present study, boys took significantly around 2,500 more steps/d than girls (12,578 vs. 10,031). The difference was also similar with the results from most studies reporting elementary school boys having higher step counts than elementary school girls in other countries^6^^,^ ^11^^,^ ^28^^,^ ^29^. In the United states, urban elementary school boys took significantly around 4,000 more steps/d than girls (16,421 vs. 12,332)^28^. McCormack *et al*.^29^ found that urban elementary school boys took significantly around 1,500 more steps/d than girls (12,270 vs. 10,081). Likewise, in Japan, Itoi *et al*.^11^ reported that urban elementary school boys took significantly around 1,500 more steps/d than girls (12,237 vs. 10,748). On the other hand, in Korea, a similar population of elementary school boys had similar steps as girls (11,165 vs. 10,708)^6^. The lack of the difference might be due to significantly higher PAL (measured by accelerometer) in girls than in boys^6^. 

In the present study, a significant relationship between steps/d and PAL was observed among children (r = 0.47, p < 0.01). In a study of Bonomi *et al*.^30^ investigating the relationship between DLW-measured PAL and individuals’ activity behavior among adults, walking was positively associated with PAL. This implies that an efficient way to increase PAL is to spend relatively more time walking. Our association data between steps/d and PAL showed that around 2,500 steps/d are needed to achieve an increased 0.1 PAL for normal-weight elementary school children. Thus, it has been suggested that elementary school children should spend more time playing outdoors and walking to school to increase PAL^11^. 

We previously reported that a significant relationship between steps per day and DLW-measured PAL was also observed only in male adults^18^. Similarly, the present study showed that a significant relationship between steps per day and PAL was observed in boys, but not in girls. In the study of Bonomi *et al*.^30^, the High PAL group (PAL > 1.75) spent significantly more time actively standing than the Low PAL group (PAL < 1.75). Time spent in active standing, defined to represent human movements performed in the standing position not related to ambulation, occupied almost 27% of the day (42% of waking hours). Tudor-Locke *et al*.^28^ also reported that elementary school girls took significantly less steps during release time (e.g., recess Δ= 479 steps, lunchtime Δ = 608 steps, and after-school Δ = 1872 steps) during the schoolday than boys. We speculate that girls who have the same PAL level as boys in the present study might have expended more energy through non-locomotive activities such as active standing during release time. This issue should be examined in detail in a further study. 

The strength of our study was the combination of the DLW method and accelerometry, enabling us to measure PAL and step counts more reliably. On the other hand, we have limitations that the sample size was so small and all of the participants were from only two elementary schools of two cities. Therefore, the results may not be applicable to all elementary school children populations in Korea. A more comprehensive randomized survey is needed to generalize our results. 

In conclusion, although DLW-measured PAL did not differ between normal-weight boys and girls, boys had a larger number of steps per day compared with girls. In addition, a positive relationship between steps/d and PAL exists in boys, but not in girls. Considerable variability may exist in the relationship between the number of steps per day and PAL in elementary school girls. Therefore, locomotive activity may be the main contributor to individual PAL differences for elementary school boys, while non-locomotive activity may be the main contributor for elementary school girls. 
